# Breeding system, shell size and age at sexual maturity affect sperm length in stylommatophoran gastropods

**DOI:** 10.1186/s12862-016-0661-9

**Published:** 2016-04-29

**Authors:** Dénes Schmera, Julia Pizá, Ellen Reinartz, Sylvain Ursenbacher, Bruno Baur

**Affiliations:** Department of Environmental Sciences, Section of Conservation Biology, University of Basel, St. Johanns-Vorstadt 10, 4056 Basel, Switzerland; MTA Centre for Ecological Research, Balaton Limnological Institute, Klebelsberg Kuno 3, 8237 Tihany, Hungary; Departamento de Biología, Bioquímica y Farmacia, Laboratorio de Zoología de Invertebrados 1, Universidad Nacional del Sur, San Juan 670, 8000 Bahía Blanca, Argentina

**Keywords:** Comparative approach, Gastropods, Phylogeny, Sexual selection, Sperm competition, Sperm evolution

## Abstract

**Background:**

Sperm size and quality are key factors for fertilization success. There is increasing empirical evidence demonstrating that sperm form and function are influenced by selective pressures. Theoretical models predict that sperm competition could favour the evolution of longer sperm. In hermaphrodites, self-fertilizing species are expected to have shorter sperm than cross-fertilizing species, which use sperm stored from several mating partners for the fertilization of their eggs and thus are exposed to intense sperm competition. We tested this hypothesis by comparing original data on sperm length in 57 species of simultaneously hermaphroditic stylommatophoran gastropods from Europe and South America with respect to the species’ breeding system. We used *28S* rRNA nuclear and *COI* mitochondrial sequence data to construct a molecular phylogeny. Phylogenetic generalized linear models were applied to examine the potential influence of morphological and life-history characters.

**Results:**

The best-fit model revealed that the breeding system and age at sexual maturity influence sperm length in gastropods. In general, species with predominant cross-fertilization had longer sperm than species with predominant self-fertilization or a mixed breeding system. Across species with shells (snails), sperm length also increased with shell size.

**Conclusions:**

Our study provides evidence that sperm length in stylommatophoran gastropods is influenced by the risk of sperm competition, as well as by age at sexual maturity and shell size. This finding extends present knowledge of sperm evolution to a group of so far poorly studied simultaneous hermaphrodites.

**Electronic supplementary material:**

The online version of this article (doi:10.1186/s12862-016-0661-9) contains supplementary material, which is available to authorized users.

## Background

There exists enormous variation in the size of sperm across the animal kingdom, ranging from 0.008 mm in the wasp *Meteorus* sp. (Hymenoptera) [1] to 58 mm in *Drosophila bifurca* [2]. Sperm size and quality are key factors for fertilization success [3–5]. Yet, the adaptive significance of variation in sperm size remains poorly understood [3]. Sperm size is expected to be selected both by sperm competition and the fertilization environment [6, 7], e.g., the female reproductive tract in the case of internally fertilizing species. In taxa with sperm storage organs, sperm length may determine the ability to reach the storage organs first and to move to the ovum from the storage organs once ovulation takes place [8, 9].

Theory predicts that sperm size can increase with sperm competition risk when longer sperm achieve a higher fertilization success than shorter sperm [10, 11]. However, empirical results for internal fertilizers are conflicting. Positive relationships between sperm length and sperm competition risk have been found across nematodes [12], butterflies [13], moths [14], and frogs [15], but not in Scathophagidae (flies) [16], while the results from studies of birds vary among taxa [17]. In mammals, larger species exhibit stronger selection on sperm number than on sperm length compared to smaller species [18]. As in most taxa, our understanding of how sperm competition influences sperm size and structure is hampered by lack of understanding of sperm function [4, 19].

Stylommatophoran gastropods (land snails and slugs) show a huge variation in sperm length and structure, which is frequently used as a taxonomic character [20–24]. Sperm are monomorphic in all species so far examined, except in the slug *Arion ater,* which produces eupyrene and apyrene sperm [25]. The adaptive significance of the interspecific variation in gastropod sperm length, however, has not been examined.

All stylommatophoran gastropods are simultaneous hermaphrodites and numerous species reproduce predominantly by cross-fertilization [26–28]. Available evidence indicates that these gastropods copulated with different mating partners (e.g., 2–6 times per year in *Helix pomatia* [29], 2–7 times in *Cornu aspersum* [30]), resulting in multiple paternity in egg batches [31, 32], with 2–7 contributing fathers [33, 34]. Self-fertilization is also widespread, while other species have a mixed breeding system [35–37]. Within species, however, geographic and age-dependent variation in frequency of self-fertilization might occur [36, 38]. Thus, some species reproduce predominantly by self-fertilization [36–38].

Cross-fertilizing species store the sperm received in the spermatheca, which has a complex structure with several tubules and functions in the context of sperm competition [32, 39–41]. Sperm from different mating partners can be stored for months or even years before being used to fertilize eggs [42]. During copulation, sperm masses or spermatophores containing spermatozoa are reciprocally transferred into the vagina of the partner [43]. The spermatophore is transported in the reproductive tract of the recipient towards the bursa copulatrix, where it is eventually digested. During a relatively short period sperm leave the spermatophore and travel up the spermoviduct to reach the spermatheca, where they are stored until fertilization [44]. The vast majority of sperm (99.98 % in *Cornu aspersum* [45]), however, is transferred into the bursa copulatrix. Sperm stored with their heads in tight contact with the epithelial walls of the spermatheca survive best [46]. Rogers and Chase [47] suggested that the unified beating of the flagella of sperm stored from the first mate could provide paternity assurance through increased resistance to incoming sperm from subsequent mates, with longer and more numerous sperm resulting in a stronger resistive force [48]. Thus, sperm of cross-fertilizing species with multiple mating and sperm storage are exposed to intense sperm competition. In contrast, self-fertilizing individuals do not store any sperm from mating partners. Instead, they use for fertilization their own sperm passing the ova in the fertilization pouch [37, 49]. Sperm competition is absent in species reproducing exclusively by self-fertilization and strongly reduced in species reproducing predominantly by self-fertilization. We therefore hypothesize that stylommatophoran species with predominant cross-fertilization have longer sperm than species that reproduce exclusively or frequently by self-fertilization.

Sperm size, however, can also be influenced by the fertilization environment [6, 7]. There is a huge variability in the complexity of the sperm-storage organ in stylommatophoran gastropods ranging from a simple spermatheca consisting of one tubule to highly structured spermatheca with multiple tubules, while other species do not possess a sperm-storage organ [41, 43]. A comparative study showed that carrefour length (total length of spermatheca and fertilization pouch) in 17 gastropod species possessing a spermatheca was positively correlated with sperm length [41]. This suggests that sperm length in gastropods may not only be influenced by sperm competition but also by the morphology of the female reproductive tract.

In this study, we used original data on total sperm length from 57 terrestrial gastropod species occurring in Europe and South America and literature data on their breeding system to test the hypothesis that stylommatophoran species with cross-fertilization have longer sperm than species with self-fertilization. We used *28S* rRNA nuclear and *COI* mitochondrial sequence data to construct a molecular phylogeny. Some of the among-species variation in sperm length may be explained by allometry [7]. We therefore examined the effect of shell size across land snail species, taking into account phylogenetic inertia. Furthermore, large interspecific differences in morphology, anatomy, physiology, behaviour, egg morphology and the fertilization environment may confound results of among-species comparisons [50]. We used phylogenetical generalized linear models to explore how the breeding system, age at sexual maturity, lifespan, mode of reproduction (oviparous vs. ovoviviparous) and habitat preference might explain sperm length in 57 stylommatophoran species.

## Results

### Interspecific variation in sperm traits

The gastropod species examined differed significantly in total sperm length with mean values ranging from 101.4 μm to 1340.9 μm (Table 1; *F*_56,94_ = 3093.3, *p* < 0.001). Similarly, the species differed in the mean head length of their sperm (range: 5.6–13.8 μm; Table 1; *F*_56,94_ = 48.9, *p* < 0.001). Sperm head length was correlated with total sperm length in the species examined (*r* = 0.77, *n* = 57, *p* < 0.001). Sperm head length expressed as percentage of total sperm length varied across species from 0.9 to 7.6 % (Table 1; *F*_56,94_ = 280.3, *p* < 0.001). Considering only snails, the relative sperm length (total sperm length divided by the maximum shell dimension of the sperm donor) varied from 1.3 to 13.2 % among species (Table 1; *F*_49,94_ = 280.3, *p* < 0.001).

**Table 1:** Sperm characteristics of the terrestrial gastropod species examined

Family	Species	Total sperm length (μm)	Sperm head length (μm)	Sperm head length in % of total sperm length	Relative sperm length (in % of maximum shell dimension)
Succineidae	*Succinea putris* (Linnaeus 1758)	478.8 ± 2.47	9.3 ± 0.13	1.9 ± 0.03	2.9 ± 0.02
Chondrinidae	*Chondrina avenacea* (Bruguière 1792)	374.6 ± 5.23	6.0 ± 0.40	1.6 ± 0.09	6.3 ± 0.07
	*Chondrina clienta* (Westerlund 1883)	336.6 ± 1.41	5.6 ± 0.34	1.7 ± 0.09	5.7 ± 0.02
	*Abida secale* (Draparnaud 1801)	375.1 ± 2.71	6.7 ± 0.31	1.8 ± 0.08	5.4 ± 0.04
Lauriidae	*Lauria cylindracea* (Da Costa 1778)	474.2 ± 4.01	11.7 ± 0.17	2.5 ± 0.05	13.2 ± 0.11
Orculidae	*Orcula dolium* (Draparnaud 1801)	431.9 ± 4.68	9.5 ± 0.17	2.2 ± 0.06	6.1 ± 0.06
Pyramidulidae	*Pyramidula pusilla* (Vallot 1801)	308.0 ± 7.46	8.6 ± 0.40	2.8 ± 0.07	13.2 ± 0.27
Vertiginidae	*Vertigo pygmaea* (Draparnaud 1801)	207.9 ± 1.31	8.8 ± 0.49	4.2 ± 0.24	11.3 ± 0.06
	*Columella columella* (Martens 1830)	176.6 ± 5.29	8.9 ± 0.43	5.0 ± 0.18	6.2 ± 0.18
Enidae	*Ena montana* (Draparnaud 1801)	590.7 ± 6.41	11.8 ± 0.23	2.0 ± 0.06	4.2 ± 0.04
Clausiliidae	*Clausilia rugosa* (Draparnaud 1801)	689.5 NA	8.2 NA	1.2 NA	9.9 NA
	*Clausilia bidentata* (Strøm 1765)	781.8 ± 1.15	7.5 ± 1.14	1.0 ± 0.02	7.4 ± 0.01
	*Macrogastra plicatula* (Draparnaud 1801)	1209.8 NA	13.0 NA	1.1 NA	9.1 NA
	*Macrogastra ventricosa* (Draparnaud 1801)	1195.4 NA	13.2 NA	1.1 NA	6.2 NA
	*Cochlodina laminata* (Montagu 1803)	1040.3 ± 1.47	10.7 ± 0.21	1.0 ± 0.02	7.1 ± 0.01
	*Cochlodina fimbriata* (Rossmässler 1835)	1119.5 ± 5.94	10.1 ± 0.28	0.9 ± 0.03	7.4 ± 0.04
	*Balea perversa* (Linnaeus 1758)	751.7 ± 0.45	8.7 ± 0.33	1.2 ± 0.05	8.5 ± 0.01
	*Balea biplicata* (Montagu 1803)	1061.9 ± 10.95	12.6 ± 0.19	1.2 ± 0.02	6.2 ± 0.07
Bothriembryontidae	*Discoleus aguirrei* (Doering 1884)	1215.9 ± 11.64	11.3 ± 0.50	0.9 ± 0.05	6.4 ± 0.05
	*Discoleus ameghinoi* (von Ihering 1908)	1232.6 ± 10.73	10.7 ± 0.50	0.9 ± 0.05	5.9 ± 0.05
Odontostomidae	*Plagiodontes patagonicus* (d’Orbigny 1835)	1340.9 ± 11.48	13.8 ± 0.15	1.0 ± 0.02	6.7 ± 0.05
	*Cyclodontina (Ventania) avellanedae* (Doering 1881)	908.0 ± 11.36	12.6 ± 0.37	1.4 ± 0.04	4.1 ± 0.08
Strophocheilidae	*Austroborus lutescens dorbignyi* (Doering 1876)	1050.7 ± 5.68	11.8 ± 0.30	1.1 ± 0.03	3.5 ± 0.02
Discidae	*Discus rotundatus* (Müller 1774)	429.4 ± 3.48	9.0 ± 0.17	2.1 ± 0.02	7.6 ± 0.05
Oxychilidae	*Oxychilus navarricus helveticus* (Blum 1881)	101.4 ± 0.65	7.7 ± 0.11	7.6 ± 0.16	1.3 ± 0.01
	*Oxychilus draparnaudi* (Beck 1837)	188.9 ± 1.61	8.1 ± 0.02	4.3 ± 0.03	1.8 ± 0.01
	*Aegopinella nitens* (Michaud 1831)	103.6 ± 2.29	7.6 ± 0.39	7.3 ± 0.33	1.2 ± 0.02
Zonitidae	*Zonitoides nitidus* (Müller 1774)	185.3 ± 3.96	9.3 ± 0.10	5.0 ± 0.10	3.2 ± 0.06
Limacidae	*Limax maximus* Linnaeus 1758	242.4 ± 1.52	8.1 ± 0.40	3.4 ± 0.14	–
	*Limax tenellus* Müller 1774	160.2 ± 1.46	6.3 ± 0.07	3.9 ± 0.08	–
	*Limax cinereoniger* Wolf 1803	289.7 ± 0.66	8.4 ± 0.15	2.9 ± 0.06	–
Agriolimacidae	*Deroceras reticulatum* (Müller 1774)	119.5 ± 0.59	7.9 ± 0.10	6.6 ± 0.06	–
Vitrinidae	*Vitrina pellucida* (Müller 1774)	201.2 NA	5.8 NA	2.9 NA	6.7 NA
	*Vitrinobrachium breve* (Férussac 1821)	267.0 NA	7.6 NA	2.9 NA	5.0 NA
Arionidae	*Arion (ater) rufus* (Linnaeus 1758)	327.8 ± 3.99	7.0 ± 0.22	2.1 ± 0.09	–
	*Arion vulgaris* (Moquin-Tandon 1855)	340.7 NA	6.6 NA	1.9 NA	–
	*Arion distinctus* (Mabille 1868)	366.3 NA	6.6 NA	1.8 NA	–
Helicidae	*Helix pomatia* Linnaeus 1758	1007.8 ± 11.43	12.8 ± 0.34	1.3 ± 0.02	2.4 ± 0.03
	*Cepaea nemoralis* (Linnaeus 1758)	744.2 ± 2.15	10.7 ± 0.52	1.4 ± 0.07	3.1 ± 0.01
	*Cepaea hortensis* (Müller 1774)	767.8 ± 5.41	10.9 ± 0.21	1.4 ± 0.03	4.1 ± 0.03
	*Cepaea vindobonensis* (Férrusac 1821)	1180.5 ± 11.92	13.8 ± 0.47	1.2 ± 0.05	5.3 ± 0.05
	*Cornu aspersum* (Müller 1774)	671.6 ± 6.15	10.5 ± 0.08	1.6 ± 0.003	2.0 ± 0.02
	*Eobania vermiculata* (Müller 1774)	1071.1 ± 11.31	11.1 ± 0.48	1.0 ± 0.05	3.7 ± 0.04
	*Theba pisana* (Müller 1774)	763.2 ± 2.68	8.5 ± 0.07	1.1 ± 0.01	4.1 ± 0.01
	*Arianta arbustorum* (Linnaeus 1758)	847.9 ± 5.40	9.6 ± 0.16	1.1 ± 0.03	5.1 ± 0.03
	*Helicigona lapicida* (Linnaeus 1758)	614.2 ± 3.83	7.1 ± 0.08	1.2 ± 0.02	3.8 ± 0.02
	*Isognomostoma isognomostomos* (Schröter 1784)	634.0 ± 3.32	9.9 ± 0.17	1.6 ± 0.02	7.1 ± 0.03
Bradybaenidae	*Fruticicola fruticum* (Müller 1774)	337.7 ± 6.42	9.8 ± 0.06	2.9 ± 0.04	1.8 ± 0.03
Cochlicellidae	*Cochlicella acuta* (Müller 1774)	332.7 ± 4.10	5.7 ± 0.13	1.7 ± 0.06	2.4 ± 0.02
Helicodontidae	*Helicodonta obvoluta* (Müller 1774)	610.1 ± 3.60	7.2 ± 0.29	1.2 ± 0.04	4.9 ± 0.03
Hygromiidae	*Helicella itala* (Linnaeus 1758)	320.0 ± 3.58	8.0 ± 0.12	2.5 ± 0.01	2.5 ± 0.02
	*Candidula intersecta* (Poiret 1801)	248.3 NA	7.6 NA	3.1 NA	2.1 NA
	*Xerolenta obvia* (Menke 1828)	313.4 ± 3.67	8.9 ± 0.07	2.9 ± 0.06	5.5 ± 0.02
	*Monachoides incarnatus* (Müller 1774)	490.4 NA	7.3 NA	1.5 NA	3.6 NA
	*Trochulus villosus* (Studer 1789)	345.0 ± 1.60	7.9 ± 0.08	2.3 ± 0.02	2.7 ± 0.01
	*Trochulus sericeus* (Draparnaud 1801)	319.5 ± 0.63	7.3 ± 0.01	2.3 ± 0.01	3.5 ± 0.01
	*Monacha cartusiana* (Müller 1774)	347.4 ± 0.33	7.0 ± 0.31	2.0 ± 0.09	2.3 ± 0.002

Mean values ± s.e. are presented. s.e. indicate inter-individual variation. *NA* not applicable (only one individual examined). Sample sizes are given in Additional file 3

### Phylogenetic tree and mode of evolution

**Fig. 1:**
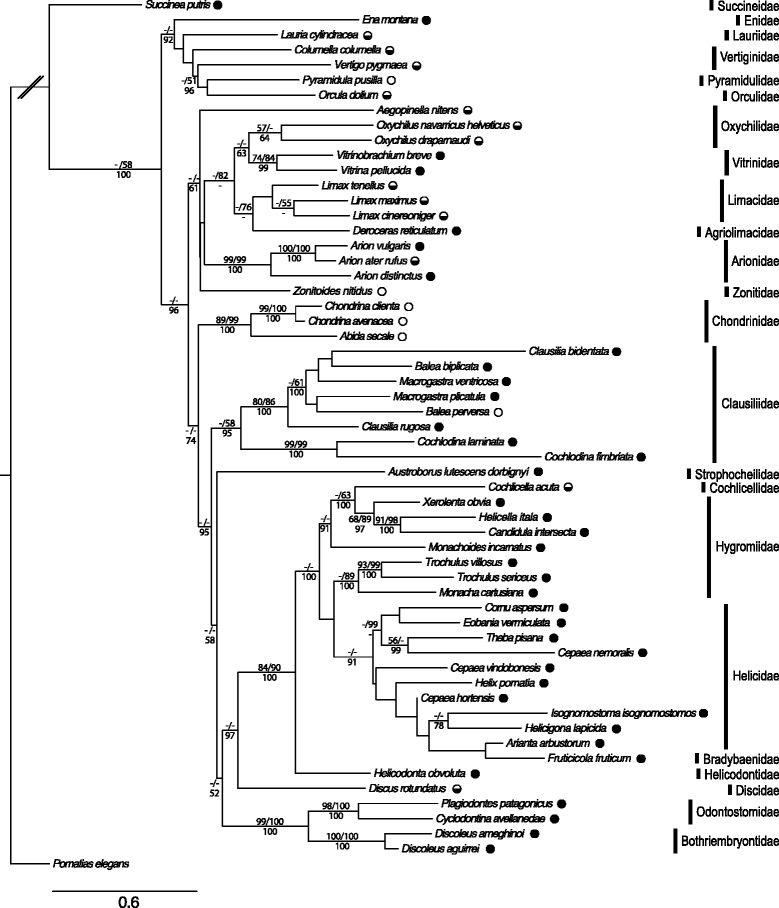
Maximum likelihood tree of the concatenated sequences (*28S* and *COI*). The tree was obtained using PhyML v3.0 [97]. Sequences of 58 terrestrial gastropod species (the prosobranch *Pomatias elegans* served as outgroup) were considered. Values of bootstrap support higher than 50 % are shown for Maximum Parsimony (higher left), Maximum Likelihood (higher right) and Bayesian Inference (lower). Families and the breeding system are indicated (full circle: predominant cross-fertilization; half empty circle: mixed reproductive strategy; empty circle: predominant self-fertilization)

The Maximum-Likelihood (ML) reconstruction for the combined data (both gene sections) was conducted with the best-fit model of substitution using JModelTest. The best fit model was TVM + I + G (freq. A = 0.4042; freq. C = 0.1691; freq. G = 0.1510; freq. T = 0.2757; R(a) = 0.4471; R(b) = 3.8949; R(c) = 0.7740; R(d) = 1.8532; R(e) = 3.8949; R(f) = 1; proportion of invariable sites = 0.3650; gamma distribution shape parameter = 0.3530). The ML analysis resulted in a relatively well-resolved topology (Fig. 1) and the Bayesian inference analysis (BI) produced very similar topologies.

The phylogenetic relationships of the different families were not well resolved (no bootstrap support in most cases; Fig. 1). However, most families were monophyletic. In those families, which appear not to be monophyletic, the bootstrap support was limited.

The occurrence of predominant self-fertilization and/or a mixed reproductive system was found in several distantly related families (e.g., Arionidae, Clausiliidae, Limacidae). The analysis of the ancestral state reconstruction using BayesTraits revealed that self-fertilization is the ancestral state (probability = 99.66 %). This suggests a very old and common origin (not necessarily used by all current species) and implies that at least five origins of mixed mating systems, two origins of self-fertilization and one loss of self-fertilization (a total of 8 transitions) are needed to explain the current status of the species analysed.

For the variables shell type, breeding system, mode of reproduction, age at sexual maturity, lifespan and habitat specificity, Pagel’s *λ* ranged from 0.88 to 0.93 when all gastropod species were considered (Additional file 1), indicating some phylogenetic dependence of the traits. Considering only snails, *λ* ranged from 0.87 to 0.93 (Additional file 1). In all cases, *λ* was significantly different from both 0 and 1, suggesting an evolution model that is different from Brownian motion.

### Effects of breeding system and life-history traits

**Fig. 2:**
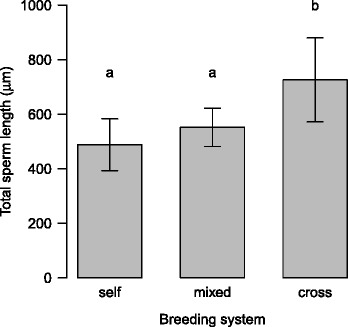
Effect of the breeding system on sperm length in gastropods. Phylogenetic effects and the influence of age at sexual maturity have been taken into account. Bars indicate mean values, whiskers standard errors. Different letters indicate significant differences between groups with different breeding systems (Tukey test, *p*-value adjusted following Westfall [115]). Sample sizes are 6, 13 and 38 from left to right

**Fig. 3:**
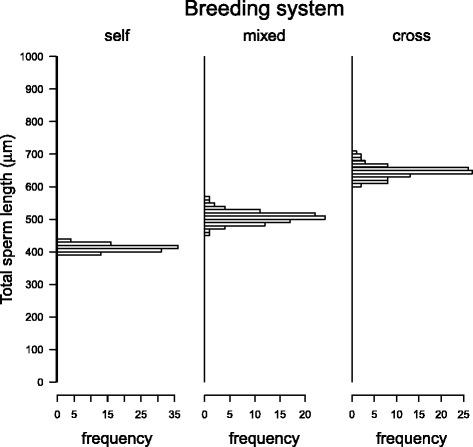
Effect of phylogenetic uncertainty on how the breeding system influences total sperm length in gastropods. Histograms represent the frequency distribution of the coefficients in response to different phylogenetic trees

**Fig. 4:**
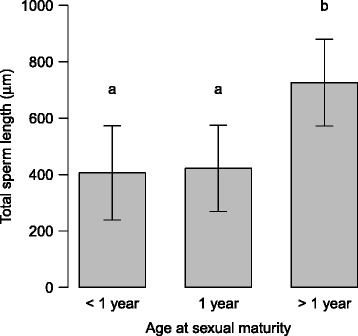
Effect of age at sexual maturity on sperm length in gastropods. Phylogenetic effects and the influence of the breeding system have been taken into account. Bars indicate mean values, whiskers standard errors. Different letters indicate significant differences between age groups (Tukey test, *p*-value adjusted following Westfall [115]). Sample sizes are 5, 18 and 34 from left to right

**Fig. 5:**
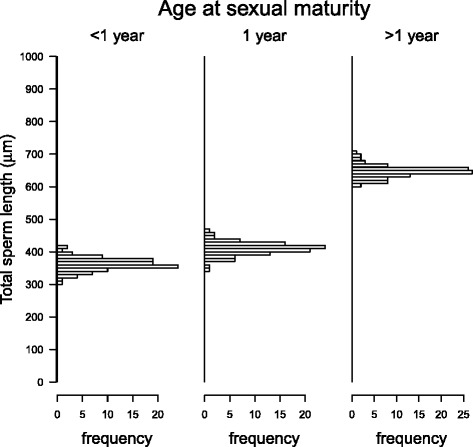
Effect of phylogenetic uncertainty on how age at sexual maturity influences total sperm length in gastropods. Histograms represent the frequency distribution of the coefficients in response to different phylogenetic trees

Considering phylogenetic information of the gastropod species examined, the best-fit model (with the lowest AICc) revealed that sperm length was affected by both the breeding system and the age at sexual maturity (Tables 2 and 3). Delta AICc values and Akaike weights did not support any alternative model (Tables 2 and 3). The most likely model (Gast1) showed that gastropod species with predominant cross-fertilization had longer sperm than species with a mixed breeding system and species with predominant self-fertilization (Fig. 2). Phylogenetic uncertainty had only a minor effect on this pattern (Fig. 3). The most likely model also showed that species whose individuals need more than one year to attain sexual maturity had significantly longer sperm than other gastropod species whose individuals reach sexual maturity earlier (Fig. 4). Phylogenetic uncertainty had only a small effect on this pattern (Fig. 5).

**Table 2:** Best fit phylogenetic generalized linear models (∆AICc < 3) explaining sperm length in gastropods

Model	Model specification	AICc	∆AICc	Weight
Gast1	Sperm length ~ 1 + Age + Breeding	773.0	0.00	0.456
Gast2	Sperm length ~ 1 + Age + Breeding + Shell type	775.2	2.19	0.152
Gast3	Sperm length ~ 1 + Age	775.5	2.45	0.134
Gast4	Sperm length ~ 1 + Age + Breeding + Lifespan	775.5	2.50	0.131
Gast5	Sperm length ~ 1 + Age + Breeding + Reproduction	775.6	2.56	0.127

Age: age at sexual maturity; Breeding: breeding system; Reproduction: reproductive mode

Sample size: 57 species

**Table 3:** ANOVA tables of the best fit phylogenetic generalized linear models using Type III sums of squares explaining sperm length in gastropods

Model	Predictor	df	Sum Sq	Mean Sq	*F*	*p*
Gast1	Age	2	3556.5	1778.2	10.48	<0.001
	Breeding	2	1530.1	765.1	4.510	0.016
	Residuals	52	9623.1	185.1		
Gast2	Age	2	1567.2	783.6	5.532	0.006
	Breeding	2	1672.3	836.1	5.903	0.005
	Shell type	3	761.4	253.8	1.792	0.161
	Residuals	49	9383.1	191.5		
Gast3	Age	2	4697.3	2348.7	10.270	<0.001
	Residuals	54	12349.6	228.7		
Gast4	Age	2	2074.4	1037.2	5.995	0.004
	Breeding	2	1530.5	765.2	4.423	0.017
	Lifespan	1	0.3	0.3	0.002	0.965
	Residuals	51	11108.9	217.8		
Gast5	Age	2	3430.0	1714.9	9.969	<0.001
	Breeding	2	1140.1	570.1	3.314	0.044
	Reproduction	1	7.9	7.9	0.046	0.831
	Residuals	51	10085.3	197.8		

Age: age at sexual maturity; Breeding: breeding system; Reproduction: reproductive mode

**Fig. 6:**
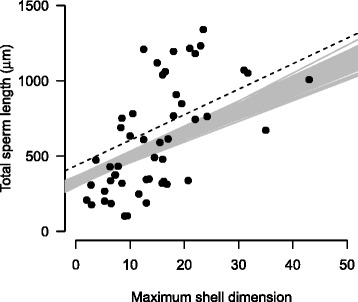
Relationship between total sperm length and maximum shell dimension in snails (*n* = 50 species). The dotted line represents the cross-species regression corrected for the most likely phylogeny, while grey lines represent alternatives phylogenetic models

Considering exclusively phylogenetic information of the snail species examined, several models showed that maximum shell dimension had a strong impact on sperm length (Tables 4 and 5). The most likely model (Snail1) revealed that sperm length of snails was affected by maximum shell dimension and age at sexual maturity (Tables 4 and 5). This model suggests that total sperm length increases with increasing shell size and that the relationship is only weakly influenced by phylogenetic uncertainty (Fig. 6). Other, still very likely models (Snail2, Snail3 and Snail 4) included also effects of the breeding system, habitat specificity and reproductive mode (Tables 4 and 5).

**Table 4:** Best fit phylogenetic generalized linear models (∆AICc < 3) explaining sperm length in snails

Model	Model specification	AICc	∆AICc	Weight
Snail1	Sperm length ~ 1 + Age + Max	674.4	0.00	0.266
Snail2	Sperm length ~ 1 + Age + Breeding + Max	674.9	0.42	0.216
Snail3	Sperm length ~ 1 + Age + Habitat + Max	676.0	1.52	0.124
Snail4	Sperm length ~ 1 + Age + Max + Reproduction	676.4	1.92	0.102
Snail5	Sperm length ~ 1 + Breeding + Habitat + Max + Shape	676.8	2.36	0.082
Snail6	Sperm length ~ 1 + Age + Lifespan + Max	676.9	2.43	0.079
Snail7	Sperm length ~ 1 + Age + Habitat + Max + Shape	677.2	2.81	0.065
Snail8	Sperm length ~ 1 + Age + Breeding + Max + Shape	677.3	2.82	0.065

Age: age at sexual maturity; Breeding: breeding system; Habitat: habitat specificity; Max: maximum shell dimension; Shape: shell shape; Reproduction: Reproductive mode

Sample size: 50 species

**Table 5:** ANOVA tables of the best fit phylogenetic generalized linear models using Type III sums of squares explaining sperm length in snails

Model	Predictor	df	Sum Sq	Mean Sq	*F*	*p*
Snail1	Age	2	2147.4	1073.7	7.292	0.002
	Max	1	2357.7	2357.7	16.012	<0.001
	Residuals	46	6773.5	147.3		
Snail2	Age	2	1665.6	832.8	6.372	0.004
	Breeding	2	572.2	286.1	2.188	0.124
	Max	1	1479.6	1479.6	11.320	0.002
	Residuals	44	9910.3	225.2		
Snail3	Age	2	2286.7	1143.3	8.242	<0.001
	Habitat	2	450.8	225.4	1.625	0.209
	Max	1	2252.4	2252.4	16.238	<0.001
	Residuals	44	8680.5	197.3		
Snail4	Age	2	2194.2	1097.1	7.525	0.001
	Max	1	1875.2	1875.2	12.862	<0.001
	Reproduction	1	74.1	74.1	0.509	0.479
	Residuals	44	9641.5	219.1		
Snail5	Breeding	2	878.6	439.3	3.874	0.029
	Habitat	2	705.1	352.6	3.087	0.056
	Max	1	1836.6	1836.6	16.085	<0.001
	Shape	2	1840.0	920.0	8.058	0.001
	Residuals	42	7224.4	172.0		
Snail6	Age	2	1390.2	695.1	4.636	0.015
	Lifespan	1	6.4	6.4	0.042	0.837
	Max	1	2340.7	2340.7	15.613	<0.001
	Residuals	45	10214.3	226.9		
Snail7	Age	2	878.5	439.3	3.573	0.037
	Habitat	2	677.6	338.8	2.756	0.075
	Max	1	1942.7	1942.7	15.803	<0.001
	Shape	2	474.9	237.4	1.932	0.157
	Residuals	42	9000.9	214.3		
Snail8	Age	2	666.8	333.4	2.829	0.070
	Breeding	2	661.6	330.8	2.807	0.072
	Max	1	1128.8	1128.2	9.578	0.003
	Shape	2	336.6	168.3	1.428	0.251
	Residuals	42	9854.4	234.6		

Age: age at sexual maturity; Breeding: breeding system; Habitat: habitat specificity; Max: maximum shell dimension; Shape: shell shape; Reproduction: Reproductive mode

In gastropods, none of the proposed models received strong support for determining sperm head length (Table 6). The most likely candidate models included effects of age at sexual maturity, breeding system, lifespan, reproductive mode and shell type. In snails, the three most likely models indicate the importance of maximum shell size for determining sperm head length (Table 7).

**Table 6:** Best fit phylogenetic generalized linear models (∆AICc < 3) explaining sperm head length in gastropods

Model	Model specification	AICc	∆AICc	Weight
SpermHGast1	Head length ~ 1 + Lifespan	235.2	0.00	0.143
SpermHGast2	Head length ~ 1 + Age	235.6	0.43	0.116
SpermHGast3	Head length ~ 1 + Breeding + Shell type	235.6	0.46	0.114
SpermHGast4	Head length ~ 1 + Age + Breeding + Shell type	236.3	1.07	0.084
SpermHGast5	Head length ~ 1 + Age + Breeding	236.4	1.21	0.078
SpermHGast6	Head length ~ 1 + Breeding + Lifespan + Shell type	236.4	1.23	0.078
SpermHGast7	Head length ~ 1 + Age + Reproduction	236.7	1.49	0.069
SpermHGast8	Head length ~ 1 + Lifespan + Reproduction	236.7	1.55	0.066
SpermHGast9	Head length ~ 1	236.9	1.68	0.062
SpermHGast10	Head length ~ 1 + Breeding + Lifespan	236.9	1.75	0.060
SpermHGast11	Head length ~ 1 + Age + Reproduction + Shell type	237.2	2.03	0.052
SpermHGast12	Head length ~ 1 + Age + Lifespan	237.5	2.27	0.046
SpermHGast13	Sperm length ~ 1 + Lifespan + Reproduction + Shell type	238.0	2.86	0.034

Age: age at sexual maturity; Breeding: breeding system; Reproduction: reproductive mode

Sample size: 57 species

**Table 7:** Best fit phylogenetic generalized linear models (∆AICc < 3) explaining sperm head length in snails

Model	Model specification	AICc	∆AICc	Weight
SpermHSnail1	Head length ~ 1 + Max	200.0	0.00	0.432
SpermHSnail2	Head length ~ 1 + Lifespan + Max	201.3	1.34	0.221
SpermHSnail3	Head length ~ 1 + Max + Reproduction	202.3	2.25	0.140
SpermHSnail4	Head length ~ 1 + Age + Breeding + Shape	202.8	2.78	0.108
SpermHSnail5	Head length ~ 1 + Age + Breeding	202.9	2.92	0.100

Age: age at sexual maturity; Breeding: breeding system; Max: maximum shell dimension; Shape: shell shape; Reproduction: Reproductive mode

Sample size: 50 species

## Discussion

Our study showed that sperm length in the gastropod species examined was influenced by the breeding system and age at sexual maturity in all models. Species with cross-fertilization had longer sperm than species with self-fertilization or a mixed breeding system. In the models that considered exclusively snail species, sperm length was also affected by shell size. We used exclusively original data on sperm length measured by the same person in the same way, which excludes unexplained variation owing to different measurement techniques applied in different studies. We also present data on sperm length in tiny snails with a shell height of 1.9–3.7 mm (Table 1, Additional file 2). Surprisingly, these snails have in relation to their shell size relatively long sperm (e.g. *Vertigo pygmaea* with a shell height of 2.0 mm has on average 207.9 μm long sperm).

### Female reproductive tract

In species with internal fertilization and multiple mating, sperm size is expected to be selected both by the female reproductive tract (the fertilization environment) and by sperm competition [5, 7, 41, 48]. In a study comparing 17 terrestrial gastropod species, sperm length was found to be positively correlated with the total length of the spermatheca and fertilization pouch after controlling for differences in shell size [41]. This finding supports the suggestion that the divergence in sperm length results from sperm size evolving in response to changing female reproductive tract [51]. Indeed, experimental evolution studies showed that changing female spermatheca length can drive the divergence in sperm length in *Drosophila melanogaster* [52, 53].

In helicid land snails, the sperm received are stored in an ordered manner in the spermatheca, usually at the bulbous blind ends of the tubules and with their heads in tight contact with the spermathecal epithelium [47, 54]. It has been suggested that the beating of the flagella of sperm from the first mate could provide paternity assurance through increased resistance to incoming sperm from subsequent mates [46, 47], with longer sperm and a larger number of sperm resulting in a stronger resistive force [48]. Thus, longer sperm competing with sperm from other mates may have a fertilization advantage by occupying and/or retaining occupancy in the storage organ better than shorter sperm. In contrast, autosperm used for self-fertilization are obtained from the spermoviduct and/or hermaphrodite duct [37, 49], and thus are not stored in a spermatheca in competition with sperm from other mates. It is important to note that most species with predominantly self-fertilization do not possess a spermatheca [41, 55].

The physical characteristics of female sperm-storage organs may impose stabilizing selection on sperm length [56]. In gastropods with spermatheca and cross-fertilization, the potential of blocking the storage organ for sperm from future mates may in turn favour divergence in spermatheca length [48]. Longer storage organs could allow the female function to take up sperm from more mating partners and thereby to benefit from a greater control over the fertilization process [53, 57, 58]. This could result in a coevolution leading to the association between sperm length and the length of the female sperm-storage organ found across snail species [41, 59], as well as across other animal taxa including moths [14], flies [16, 57, 60], beetles [61], birds [62] and mammals [63].

In this context the intraspecific variation of sperm length is of interest. In stylommatophoran gastropods, data on intraspecific variation of sperm length are available for only a single species [8, 64]. In 23 populations of *Arianta arbustorum* sampled across the distributional range of the species, a difference of 11 % in total sperm length between the lowest and highest population means was found [64]. Differences among *A. arbustorum* populations explained 62.9 % of the variance in total sperm length, differences among individual snails within population 23.4 % and differences within individual snail 13.7 %. Furthermore, individuals of *A. arbustorum* showed consistent sperm length in successive matings, and mean sperm length was not correlated with the number of sperm delivered in a spermatophore [8]. A breeding experiment with offspring of *A. arbustorum* raised at three different temperatures revealed both environmental and genetic effects on sperm length [65]. The relatively small intraspecific variation in this species can be contrasted with the huge interspecific variation in sperm length found in our study with sperm of *Plagiodontes patagonicus* being 13 times longer than those of *Oxychilus navarricus helveticus* (Table 1).

### Sperm competition

Success in sperm competition is predicted to be influenced by variation in sperm and ejaculate quality and by interactions among competing sperm [5, 66, 67]. Sperm of cross-fertilizing terrestrial gastropod species are exposed to intense sperm competition due to multiple mating with different partners and long-term sperm storage [33, 42, 59]. Yet, apart from sperm number, relevant sperm characteristics are poorly understood in terrestrial gastropods.

In general, sperm size may influence the outcome of sperm competition through multiple mechanisms (reviewed in Simmons [19] and Pizzari and Parker [5]). In stylommatophoran gastropods, sperm size may influence: (i) the number of sperm delivered during a copulation because of a potential trade-off between sperm length and number; (ii) sperm longevity and/or swimming speed; and (iii) the defence ability of sperm stored in the spermatheca of the recipient against sperm from future mates (see above). Available evidence suggests that there is no trade-off between sperm length and the number of sperm delivered in a spermatophore. In none of the four *Arianta arbustorum* populations examined was a correlation between mean sperm length and the number of sperm delivered found after having removed the effect of shell size [8]. Sperm competition may select for longer sperm if sperm size is positively linked to sperm competitiveness through increased velocity, motility, longevity, and/or a better ability to defend their position in spermatheca [10, 66]. In *Drosophila melanogaster*, relatively long and slow sperm are at an advantage in entering or remaining in the “fertilization set” of the female during the sperm storage and displacement phase of sperm competition, thereby gaining a chance of being used for fertilization later [67].

Sperm length in the snail *A. arbustorum* was neither correlated with mean velocity nor with percentage motility or longevity, all measured *in vitro* [8]. However, mean sperm velocity differed among individual snails (range 52–112 μm/s). Furthermore, the percentage motility and longevity of sperm differed between snails from the two populations, but were not affected by shell size [8]. After spermatophore transfer, longer sperm might have a higher probability of leaving the spermatophore and escaping the peristaltic waves of the duct transporting them to the sperm-digesting organ [44]. In *Helix pomatia* and *Cornu aspersum*, only about 0.025 % of the transferred sperm are stored in the spermathecal tubules of the storage organ [44, 45]. However, experiments are needed to test whether longer sperm have an advantage in this process.

With the unified beating of their flagella sperm stored in the spermatheca of the recipient may generate resistance to incoming sperm from subsequent mates entering the tubules and thus provide paternity assurance [47], with longer and more numerous sperm resulting in a stronger resistive force [46, 48]. This hypothesis might also explain why sperm of stylommatophoran species with a complex sperm-storage organ are longer than those of other gastropods with simple or no sperm storage organ, most of them being frequently or predominantly self-fertilizing species. Several comparative studies considering a diverse array of taxa examined the evolutionary relationship between sperm length and some estimates of risk or intensity of sperm competition (reviewed in Pitnick et al. [7]). For example, in rhabditid nematodes, males of gonochoristic species had significantly larger sperm than males of the hermaphrodite species [12]. In nematodes, males of gonochoristic species are normally exposed to a high risk of sperm competition, whereas hermaphroditic species mainly reproduce by self-fertilization resulting in a low sperm competition risk. The main conclusion to be drawn from the various correlational studies is that sperm competition is important in the evolutionary diversification of sperm size in some but not in all animal groups.

### Allometric effects

Some of the among-species variation in sperm size may be explained by allometry: the way characters scale with shell or body size. We found a positive relationship between sperm length and shell size after having taken phylogenetic relationships into account. This finding supports the results of an earlier study on land snails with a much smaller sample size (17 species [41]) and extends our knowledge of the positive sperm length–body size relationships in other invertebrates (butterflies [13]; fruit flies [2]; nematodes [12]; an exception being dung flies [16]). Two non-mutually exclusive hypotheses have been suggested for the sperm length–body size relationships observed across some invertebrate groups [7]. First, increases in body size and sperm size may be independently favoured by sexual selection. Second, because of the energetic demands of producing relatively long sperm, sperm size and body size may coevolve. Interspecific studies with *Drosophila* have demonstrated substantial energetic costs and life history trade-offs associated with the production of relatively long sperm [2, 7, 68], while intraspecific analyses with *D. hydei* showed how larger body size mitigates those costs for males [69]. Similar studies are not available for terrestrial gastropods.

### Advantage of self-fertilization

Simultaneous hermaphroditism is advantageous when mates are hard to find. In such situations each sexually mature conspecific encountered is a potential mating partner. Simultaneous hermaphroditism also offers opportunities for self-fertilization. Evolutionary theory predicts the conditions under which simultaneous hermaphrodites should reproduce by self-fertilization [70]. Depending on the relative balance of the costs and benefits, populations are assumed to evolve towards complete cross-fertilization or complete selfing [71]. Nevertheless, mixed mating strategies are also frequently observed in a variety of taxa in nature [72, 73].

Self-fertilization provides advantages including assurance of reproduction in the absence of mating partners, preservation of highly fit genotypes, and reduced energy allocation to both sperm production and mating behaviour [70, 71]. On the other hand, self-fertilization results in low heterozygosity, which in turn reduces the chances to adapt to changing environmental conditions and thus enhances the risk of local extinction [74].

In stylommatophoran gastropods, the frequency of self-fertilization varies greatly among species and – to a smaller extent – even among populations within species [37, 38]. In some species, it is rare, in others it occurs occasionally, and still in others self-fertilization occurs regularly [75]. Low frequencies of self-fertilization also occur in species so far considered as obligate cross-fertilizers. For example, in a natural population of *A. arbustorum*, a low frequency of self-fertilization was found in two out of 41 mother-progeny arrays: Two mother snails produced 2.0 and 18.2 % of their offspring by self-fertilization, while the remaining 39 mother snails reproduced exclusively by cross-fertilization [76]. However, laboratory breedings showed strong effects of inbreeding depression in this species; selfing individuals had a reproductive success of 1–2 % compared with that of cross-fertilizing individuals [77]. Strong inbreeding depression was also observed in *Triodopsis albolabris* [78], another snail species that predominantly reproduces by cross-fertilization. In contrast, inbreeding depression appears to be absent in the slug *Deroceras agreste*, a species with frequent self-fertilization [37]. Self-fertilizing individuals were two to four times more fecund, had a longer lifespan and the growth rate of their offspring was higher than that of cross-fertilizing individuals. Similarly, inbreeding depression was very low in self-fertilizing *Balea perversa* [79, 80].

Within-species variation in breeding system could influence the results of our analyses. To minimize errors due to misclassified species, we used three categories of breeding systems: predominantly cross-fertilizing species, predominantly self-fertilizing species and species with a mixed breeding system. To the latter category we assigned all species in which the available information was ambiguous, i.e., some authors reported cross-fertilization but records on selfing were also found in the same species. Our analyses showed that the main differences in sperm length were between cross-fertilizing species with longer sperm and both self-fertilizing species and species with a mixed breeding system, which produced shorter sperm. Available information indicates that species with a mixed breeding system fertilize 70–80 % of their eggs by self-fertilization, suggesting less intense sperm competition than in cross-fertilizing species. This could be an explanation for the small difference in sperm length between species with predominant self-fertilization and species with a mixed breeding system.

## Conclusions

In conclusion, we found evidence that sperm length in stylommatophoran gastropods is influenced by the risk of sperm competition experienced in different breeding systems, as well as by age at sexual maturity and shell size. However, female morphology (the size and structure of the sperm-storage organ) may also influence divergence in sperm length. Our findings extend present knowledge of sperm evolution in a rarely studied group of simultaneous hermaphrodites and highlight the complexity of postcopulatory processes in this group.

## Methods

### Gastropod species

We analysed the sperm of 57 terrestrial gastropod species (50 land snail and 7 slug species) representing 23 families of the subclass Stylommatophora (Table 1). Adult specimens were collected at various localities in Europe and South America during the reproductive seasons in 2011 and 2012 (Additional file 3). Sampling of gastropods complied with national and international guidelines. The snails and slugs collected were neither protected by law nor endangered in the countries they were collected. The gastropods sampled were immediately frozen at −80 °C. Nomenclature of gastropod families and species follows Bouchet et al. [81] and Breure and Romero [82].

### Sperm length measurements

In stylommatophoran gastropods, the autosperm produced are stored in the midsection of the hermaphroditic duct, the vesicula seminalis [83]. We dissected out the hermaphroditic duct of three specimens per species (in a few species the sample size was smaller; Additional file 3). Using a fine needle, we opened the wall of the sperm-containing part of the hermaphrodite duct along its longitudinal axis and placed it for 12–24 h in 240 μl PBS with Ca^2+^/Mg^2+^ at 4 °C. We measured sperm length following Minoretti and Baur [8]. Aliquots of 20 μl sperm suspension were placed on two microscopic slides, covered with a coverslip and sealed with translucent nail polish. We digitized randomly chosen spermatozoa using a camera (Canon PowerShot S70) mounted on a compound microscope (Leica DMLD, PH3, magnification 40–100x) connected to a Macintosh computer. From these images, we measured total sperm length (head and tail) and sperm head length for 25 spermatozoa from each specimen using ImageJ (version 1.43f; https://imagej.nih.gov/ij/). Freezing at −80 °C does not appear to affect sperm length. Sperm obtained from freshly killed individuals of *Arianta arbustorum* (never frozen) and sperm from the same individuals kept at −80 °C for 2 months did not differ in length (N. Minoretti, unpublished data).

We assessed the reliability of multiple length measurements on the same sperm (eight sperm from one individual of eight different species on 12 days) by calculating the repeatability following Lessells and Boag [84]. Repeatability of multiple sperm length measurements was 0.97 indicating that the technique was accurate.

To adjust sperm characters to snail size, we measured shell width and shell height of each snail individual to the nearest 0.1 mm using a vernier calliper (for slug species see below).

### Gastropod characteristics

Data on the predominant breeding system (cross-fertilization, self-fertilization, or a mixed system) and reproductive mode (oviparity in species that deposit eggs or ovoviviparity in species that retain fertilized eggs in the female reproductive duct) were compiled from different sources [26–28, 36, 49]. Data on life-history traits (maximum shell dimension of adult snails, age at sexual maturity and lifespan) and habitat specificity (open-land: species exclusively occurring in open habitat; forest: species mainly found in wooded areas; ubiquitous: species found in different types of habitat) were obtained from various sources [37, 38, 85–88], B. Baur (unpubl. data) and J. Pizá (unpubl. data)] (see Additional file 2). Maximum shell dimension (shell height or shell width whatever is larger in a species) has been shown to be a reasonably good surrogate for body size in terrestrial snail species with different shell shape [86, 89].

The shape of gastropod shells (oblong, globose or depressed) is of ecological significance because of the strong associations between shell shape and the angle and nature of substrate on which the snails are active [90–92]. Species with oblong shells use vertical surfaces or burrow in soft substrate, species with depressed shells predominantly occur on horizontal surfaces, while globular species are less specific in their preferences. Shell shape of snails is frequently expressed as the maximum shell height divided by the maximum shell width (hereafter shell shape index, SI [93]). Using literature data on shell height and shell width we calculated SI for each snail species and assigned it to three classes: snails with flat or depressed shells (SI ≤ 0.65); snails with globose shells (0.65 < SI <1.35); and snails with oblong shells (SI ≥ 1.35). The classes correspond to peaks in the frequency distribution of the shell shape index reported in various terrestrial gastropod faunas [93]. In data analyses including all gastropods, we used the variable shell type, which includes the three shell shape classes of snails and as a fourth group all slug species examined (gastropods without shell).

### Phylogenetic analyses

We analysed sections of the *28S* and *COI* genes to determine the phylogenetic relationships between the gastropod species examined. Sequences of the *28S* were available on GenBank or iBOL for 34 species and those of *COI* for 36 species. To complete the dataset for both genes, we extracted total genomic DNA from the foot of a specimen from each of 32 species using QIAamp DNA Mini Kit (Qiagen, Hombrechtikon, Switzerland). The *28S* gene was amplified by PCR with the primers *28S*-forward and *28S*-reverse [41] in a 25 μl volume using Taq PCR Core kit (Qiagen) with 0.5 μM of each primer, 1.5 mM of MgCl_2_, 1x Q-solution and 1x buffer. Amplifications were conducted for 35–45 cycles (depending on the DNA quality) of 95 °C during 30 s, 50 °C during 30 s and 72 °C during 60 s. The *COI* was amplified with the primers FCOI and RCOI under the same conditions as presented above, except for MgCl_2_ (2.5 mM) [94]. The PCR was composed of 35–45 cycles of 95 °C during 45 s, 52 °C during 45 s and 72 °C during 60 s. PCR products were checked on 1.5 % agarose gel and thereafter sent to Macrogen (Seoul, Rep. of Korea) for sequencing. All sequences were deposited in GenBank (for accession nos. see Additional file 4). We used sequences of the prosobranch land snail *Pomatias elegans* from GenBank as an outgroup.

The sequences were checked using CodonCode Aligner v 4.0.3 (CodonCode Corporation, Centerville, USA) and aligned with ClustalX v2.0 [95]. We selected the appropriate model of sequence evolution using JModelTest v2.1.5 [96] based on AIC (Akaike Information Criterion) and applied to the different phylogenetic reconstruction methods. We ran Maximum Likelihood analyses (ML) using PhyML v3.0 [97]. We applied PAUP* v4.0b.10 [98] to evaluate the Maximum Parsimony (MP) trees (heuristic searches with random stepwise addition and TBR branch swapping options). The robustness of the trees was assessed by bootstrap resampling using 1000 random MP and ML repetitions. Bayesian inference analyses (BI) were performed with MrBayes v3.12 using the GTR + I + G model of substitution [99]. The analysis was run with four chains of 10^7^ generations, with a sampling every 100 generations. The first 10 % of the trees were discarded as burn-in. In order to check the stability of the simulations, the different parameters were plotted using Tracer v1.6 [100]. To evaluate the effect of phylogenetic uncertainty in the morphological analyses, 100 trees of the MrBayes analysis were sampled (one tree was selected every 91,000 generations). The ancestral state of the breeding system (self-fertilization or cross-fertilization) was examined using Multistate ML with BayesTraits v2 [101] and 100 random trees obtained from the MrBayes analysis.

### Data analyses

Relative sperm length was calculated based on the shell size of the sperm-producing individual (expressed in % of maximum shell dimension; slugs were excluded from this analysis). Relative sperm length indicates the length of sperm in relation to the size of the animal and was only used for illustrative purposes (Table 1).

For among-species comparisons of total sperm length and sperm head length, we used species mean values of life-history traits obtained from the literature (see above). The maximum shell dimension of a snail was used as a measure of body size, which allowed us to test possible effects of allometry on sperm size. Extended body length of slugs cannot be compared with any measures of shell size in snails. We therefore ran all analyses twice. First, we used data of all gastropod species (*n* = 57), but did not consider body size in the analyses. Second, we used data of all snail species (*n* = 50, excluding slug species) and considered maximum shell dimension as a measure of body size in the analyses.

Snail shell width, shell height and the derived maximum shell dimension were measured on ratio scales. For all gastropods, age at sexual maturity and lifespan were expressed on ordinal scales, while breeding system, habitat specificity and reproductive mode were expressed on nominal scales. Due to problems associated with the analyses of ordinal scale data, these data were reduced to nominal data [102]. Variance inflation factors (VIF) were used to test for collinearity of traits. Gastropod traits showed only moderate collinearity (the highest observed value was observed both for gastropods and snails reaching sexual maturity at an age of 1 year with values of 3.092 and 3.121, respectively). We therefore used all traits in the statistical modelling.

Pagel’s maximum likelihood (hereafter *λ*) was used to estimate the phylogenetic signal in our dataset. *λ* varies between 0 (phylogenetic independence of the data) and 1 (strong phylogenetic signal with a Brownian Motion evolution model). In cases of 0 < *λ* < 1, a phylogenetic dependence can be assumed. Likelihood ratio tests were used to compare estimated *λ* with values of 0 and 1 [103, 104].

We used phylogenetic generalized linear models (PGLMs) to examine how species traits explain sperm characteristics of gastropods, taking into account phylogenetic dependence of data. We considered data on age at sexual maturity, breeding system, habitat specificity, shell type and reproductive mode for gastropod species. In models considering only snails, three groups of species with different shell shape were used (depressed, globose and oblong shells), while in models considering all gastropods slugs (gastropods without shell) were considered as a fourth group in the trait shell type. We added maximum shell dimension to these traits and replaced shell type by shell shape when snail species were examined. Only main effects without interactions were examined. We assessed the performance of PGLMs based on all possible combinations of species traits. This resulted in 64–128 different models depending on whether sperm characteristics of gastropods or snails were considered. The best-fit models were selected using an information theoretic approach (following Garamszegi & Mundry [105]) based on the Akaike Information Criterion corrected for the number of cases and parameters estimated (AICc) and Akaike weights. Delta AICc indicates the difference in the fit between a particular model considered and that of the best fit model. Models with a delta AICc < 3 are shown in the Results section. AIC weight was calculated among this subset of models. Phylogenetic uncertainty was assessed by running the best statistical model with each of the 100 phylogenetic trees (see above: Phylogenetic analyses). This resulted in a frequency distribution of the particular sperm character for each predictor (Figs. 3 and 5).

Statistical analyses were run in R [106] using the packages *ape* [107], *caper* [108], *faraway* [109], *geiger* [110], *multcomp* [111], *MuMIn* [112], *nlme* [113] and *picante* [114].

### Ethics

Not applicable

### Consent to publish

Not applicable

## Additional files

Additional file 1: Pagel’s *λ* and tests of data independence (*λ* = 0) and of Brownian Motion (*λ* = 1). (a) for gastropods (*n* = 57 species, slugs included), and (b) for snails (*n* = 50 species; slugs excluded). (PDF 26 kb) Additional file 2: Shell characteristics, life-history traits and habitat specificity of the terrestrial gastropod species examined. (PDF 157 kb) Additional file 3: Gastropod species, locations of sampling, elevation, and sampling date together with sample size. (PDF 141 kb) Additional file 4: GeneBank accession numbers for the new nucleotide sequences. (PDF 9 kb)

## Abbreviations

AIC: Akaike Information Criterion
BI: Bayesian inference analysis
ML: maximum-likelihood reconstruction
PGLM: phylogenetic generalized linear model
SI: shell shape index
