# Supplementary material for: Breeding system, shell size and age at sexual maturity affect sperm length in stylommatophoran gastropods
Source: BMC Evol Biol. 2016 Apr 29;16:89. doi: 10.1186/s12862-016-0661-9 (PMC4850656; doi:10.1186/s12862-016-0661-9)
Supplement: Additional file 1: — Pagel’s λ and tests of data independence (λ = 0) and of Brownian Motion (λ = 1). (a) for gastropods (n = 57 species, slugs included), and (b) for snails (n = 50 species; slugs excluded). (PDF 26 kb) [file 12862_2016_661_MOESM1_ESM.pdf]

Additional file 1: Pagel's λ and tests of data independence (λ = 0) and of Brownian Motion (λ = 1). (a) for gastropods (n = 57 species, slugs included), and (b) for snails (n = 50 species; slugs excluded).

(a)

Trait	Observed λ	Probability of 0	Probability of 1
Shell type*	0.882	<0.001	<0.001
Breeding system	0.906	<0.001	<0.001
Mode of reproduction	0.927	<0.001	<0.001
Age at sexual maturity	0.881	<0.001	<0.001
Longevity	0.908	<0.001	<0.001
Habitat specificity	0.928	<0.001	<0.001

*three shell shape categories and slugs as an own category

(b)

Trait	Observed λ	Probability of 0	Probability of 1
Maximum shell dimension	0.873	<0.001	<0.001
Shell shape	0.875	<0.001	<0.001
Breeding system	0.898	<0.001	<0.001
Mode of reproduction	0.926	<0.001	<0.001
Age at sexual maturity	0.853	<0.001	<0.001
Longevity	0.898	<0.001	<0.001
Habitat specificity	0.927	<0.001	<0.001
