# Supplementary material for: Breeding system, shell size and age at sexual maturity affect sperm length in stylommatophoran gastropods
Source: BMC Evol Biol. 2016 Apr 29;16:89. doi: 10.1186/s12862-016-0661-9 (PMC4850656; doi:10.1186/s12862-016-0661-9)
Supplement: Additional file 2: — Shell characteristics, life-history traits and habitat specificity of the terrestrial gastropod species examined. (PDF 157 kb) [file 12862_2016_661_MOESM2_ESM.pdf]

Additional file 2: Shell characteristics, life-history traits and habitat specificity of the terrestrial gastropod species examined

Family	Species	Shell width[1] (mm)	Shell height[1] (mm)	Shell shape[2]	Breeding system[3,4]	Reproductive mode[5]	Age at sexual maturity[6]	Lifespan[7]	Habitat specificity[8]
Succineidae	*Succinea putris* (Linnaeus 1758)	8.8	16.0	1	cross	ovi	2	3	U
Chondrinidae	*Chondrina avenacea* (Bruguière 1792)	2.6	7.2	1	self	ovovivi	3	3	U
	Chondrina clienta (Westerlund 1883)	2.5	6.4	1	self	ovovivi	3	3	O
	Abida secale (Draparnaud 1801)	2.8	7.3	1	self	ovovivi	3	3	U
Lauriidae	*Lauria cylindracea* (Da Costa 1778)	1.9	3.7	1	mixed	ovovivi	3	3	U
Orculidae	*Orcula dolium* (Draparnaud 1801)	3.5	7.8	1	mixed	ovi	3	3	F
Pyramidulidae	*Pyramidula pusilla* (Vallot 1801)	2.8	1.9	2	self	ovovivi	2	3	U
Vertiginidae	*Vertigo pygmaea* (Draparnaud 1801)	1.1	2.0	1	mixed	ovi	1	2	O
	Columella columella (Martens 1830)	1.5	2.9	1	mixed	ovi	1	3	U
Enidae	*Ena montana* (Draparnaud 1801)	6.5	15.5	1	cross	ovi	3	3	U
Clausiliidae	*Clausilia rugosa* (Draparnaud 1801)	2.2	8.3	1	cross	ovi	3	3	U
	Clausilia bidentata (Strøm 1765)	2.5	10.5	1	cross	ovi	3	3	U
	Macrogastra plicatula (Draparnaud 1801)	3.0	12.5	1	cross	ovi	3	3	F
	Macrogastra ventricosa (Draparnaud 1801)	4.1	18.0	1	cross	ovi	3	3	F
	Cochlodina laminata (Montagu 1803)	3.8	16.0	1	cross	ovi	3	3	F
	Cochlodina fimbriata (Rossmässler 1835)	4.0	15.0	1	cross	ovi	3	3	F
	Balea perversa (Linnaeus 1758)	2.6	8.5	1	self	ovovivi	3	3	U
	Balea biplicata (Montagu 1803)	4.1	16.5	1	cross	ovovivi	3	3	F
Bothriembryontidae	*Discoleus aguirrei* (Doering 1884)	12.3	21.0	1	cross	ovi	3	3	O
	Discoleus ameghinoi (von Ihering 1908)	10.8	23.0	1	cross	ovi	3	3	O
Odontostomidae	*Plagiodontes patagonicus* (d'Orbigny 1835)	12.0	23.5	1	cross	ovi	3	3	O
	Cyclodontina (Ventania) avellanedae (Doering 1881)	7.1	18.5	1	cross	ovi	3	3	O
Strophocheilidae	*Austroborus lutescens dorbignyi* (Doering 1876)	21.4	31.7	1	cross	ovi	3	3	O
Discidae	*Discus rotundatus* (Müller 1774)	6.3	2.8	3	mixed	ovi	2	2	F
Oxychilidae	*Oxychilus navarricus helveticus* (Blum 1881)	9.0	4.9	3	mixed	ovi	2	3	F
	Oxychilus draparnaudi (Beck 1837)	13.0	6.5	3	mixed	ovi	2	2	U
	Aegopinella nitens (Michaud 1831)	9.5	4.8	3	mixed	ovi	2	2	F
Zonitidae	*Zonitoides nitidus* (Müller 1774)	6.5	3.7	3	self	ovi	2	2	O
Limacidae	*Limax maximus* Linnaeus 1758	170.0*		s	mixed	ovi	3	3	U
	Limax tenellus Müller 1774	40.0*		s	mixed	ovi	1	2	F
	Limax cinereoniger Wolf 1803	180.0*		s	mixed	ovi	3	3	F
Agriolimacidae	*Deroceras reticulatum* (Müller 1774)	42.0*		s	cross	ovi	1	2	U
Vitrinidae	*Vitrina pellucida* (Müller 1774)	5.3	3.0	3	cross	ovi	2	2	O
	Vitrinobrachium breve (Férussac 1821)	5.3	2.9	3	cross	ovi	2	2	F
Arionidae	*Arion (ater) rufus* (Linnaeus 1758)	120.0*		s	mixed	ovi	2	2	U

	Arion vulgaris (Moquin–Tandon 1855)	100.0*		s	cross	ovi	2	2	U
	Arion distinctus (Mabille 1868)	30.0*		s	cross	ovi	1	2	U
Helicidae	*Helix pomatia* Linnaeus 1758	43.0	43.0	2	cross	ovi	3	3	U
	Cepaea nemoralis (Linnaeus 1758)	22.0	17.0	2	cross	ovi	3	3	U
	Cepaea hortensis (Müller 1774)	18.0	13.5	2	cross	ovi	3	3	U
	Cepaea vindobonensis (Férrusac 1821)	22.0	17.1	2	cross	ovi	3	3	O
	Cornu aspersum (Müller 1774)	34.0	35.0	2	cross	ovi	3	3	U
	Eobania vermiculata (Müller 1774)	31.0	20.8	2	cross	ovi	3	3	U
	Theba pisana (Müller 1774)	24.2	18.0	2	cross	ovi	3	3	O
	Arianta arbustorum (Linnaeus 1758)	19.5	16.5	2	cross	ovi	3	3	U
	Helicigona lapicida (Linnaeus 1758)	17.0	7.9	3	cross	ovi	3	3	F
	Isognomostoma isognomostomos (Schröter 1784)	10.0	6.7	2	cross	ovi	3	3	F
Bradybaenidae	*Fruticicola fruticum* (Müller 1774)	20.7	13.8	2	cross	ovi	3	3	U
Cochlicellidae	*Cochlicella acuta* (Müller 1774)	6.2	16.2	1	mixed	ovi	2	2	O
Helicodontidae	*Helicodonta obvoluta* (Müller 1774)	12.5	5.9	3	cross	ovi	3	3	F
Hygromiidae	*Helicella itala* (Linnaeus 1758)	15.9	7.0	3	cross	ovi	2	2	O
	Candidula intersecta (Poiret 1801)	11.6	7.5	3	cross	ovi	2	2	O
	Xerolenta obvia (Menke 1828)	16.8	9.0	3	cross	ovi	2	3	O
	Monachoides incarnatus (Müller 1774)	14.5	10.7	2	cross	ovi	3	3	F
	Trochulus villosus (Studer 1789)	13.0	7.8	3	cross	ovi	2	3	F
	Trochulus sericeus (Draparnaud 1801)	8.5	5.8	2	cross	ovi	2	3	U
	Monacha cartusiana (Müller 1774)	13.5	8.0	3	cross	ovi	2	2	O

[1] Data on shell width and height were obtained from Kerney et al. [85] and J. Pizá (unpubl.). * Slugs: extended body length (in mm).

[2] Shell shape – 1: oblong; 2: globose/conical; 3: depressed; s: slug.

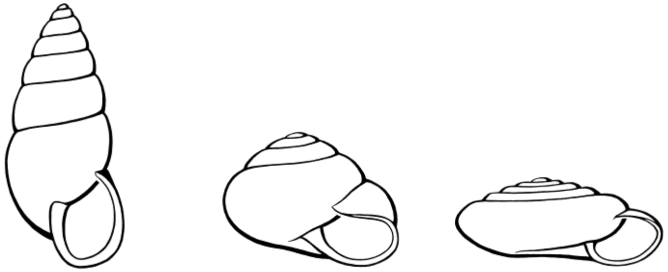

oblong globose/conical depressed

[3] Data on breeding system, reproductive and life-history traits and habitat specifity were obtained from Bengtsson & Baur [86], Baur [87], Falkner et al. [88], Heller [38], B. Baur (unpubl. data) and J. Pizá (unpubl. data).

[4] Breeding system – cross: predominant cross-fertilization; self: predominant self-fertilization; mixed: both self- and cross-fertilization.

[5] Reproductive mode – ovi: oviparous; ovovivi: ovoviviparous.

[6] Age at sexual maturity – 1: < 1 year; 2: 1 year; 3: > 1 year.

[7] Lifespan – 1: < 1 year; 2: 1–2 years; 3: > 2 years.

[8] Habitat specifity – O = openland; F = forest; U = ubiquitous.
