# Supplementary material for: Breeding system, shell size and age at sexual maturity affect sperm length in stylommatophoran gastropods
Source: BMC Evol Biol. 2016 Apr 29;16:89. doi: 10.1186/s12862-016-0661-9 (PMC4850656; doi:10.1186/s12862-016-0661-9)
Supplement: Additional file 3: — Gastropod species, locations of sampling, elevation, and sampling date together with sample size. (PDF 141 kb) [file 12862_2016_661_MOESM3_ESM.pdf]

Additional file 3: Gastropod species, locations of sampling, elevation, and sampling date together with sample size

Family	Species	Localities	Geographical coordinates	Elevation (m a.s.l.)	Sampling date	Number of gastropods	Number of sperm[1]
Succineidae	*Succinea putris* (Linnaeus 1758)	Bebenhausen, Germany	48° 34' 24.33" N, 9° 02' 04.70" E	402	09.07.2011	3	3x25
Chondrinidae	*Chondrina avenacea* (Bruguière 1792)	Chienberg bei Grindel, Switzerland	47° 23' 07.99" N, 7° 31' 26.39" E	715	11.09.2011	3	3x25
	Chondrina clienta (Westerlund 1883)	Vickleby, Sweden	56° 33' 37.32" N, 16° 27' 00.65" E	47	11.07.2011	2	2x25
	Abida secale (Draparnaud 1801)	Gempenfluh, Switzerland	47° 28' 38.93" N, 7° 38' 49.53" E	727	01.07.2012	3	3x25
Lauriidae	*Lauria cylindracea* (Da Costa 1778)	Sartrouville, France	48° 55' 52.24" N, 2° 08' 54.80" E	31	17.08.2012	3	3x25
Orculidae	*Orcula dolium* (Draparnaud 1801)	Nunningen, Switzerland<br>Wittnau, Switzerland	47° 22' 41.47" N, 7° 37' 34.92" E<br>47° 25' 54.21" N, 8° 01' 06.15" E	893<br>801	14.07.2012<br>06.08.2012	3	1x25<br>2x25
Pyramidulidae	*Pyramidula pusilla* (Vallot 1801)	Hintere Wasserfallen, Switzerland<br>Gempenfluh, Switzerland<br>Gantrisch, Switzerland	47° 22' 18.00" N, 7° 41' 44.40" E<br>47° 28' 38.93" N, 7° 38' 49.53" E<br>46° 42' 22.62" N, 7° 26' 42.04" E	1030<br>727<br>1777	16.10.2011<br>01.07.2012<br>04.08.2012	3	1x25<br>1x25<br>1x25
Vertiginidae	*Vertigo pygmaea* (Draparnaud 1801)	Torslunda, Öland, Sweden	56° 36' 57.31" N, 16° 29' 54.72" E	50	07.10.2011	3	2x25, 1x13
	Columella columella (Martens 1830)	Gantrisch, Switzerland	46° 42' 22.62" N, 7° 26' 42.04" E	1777	04.08.2012	3	1x25, 1x11
Enidae	*Ena montana* (Draparnaud 1801)	Wittnau, Switzerland	47° 25' 54.21" N, 8° 01' 06.15" E	801	06.08.2012	3	3x25
Clausiliidae	*Clausilia rugosa* (Draparnaud 1801)	Gempenfluh, Switzerland	47° 28' 38.93" N, 7° 38' 49.53" E	727	01.07.2011	1	1x25
	Clausilia bidentata (Strøm 1765)	Vickleby, Sweden	56° 34' 11.20" N, 16° 29' 42.24" E	39	11.07.2011	3	3x25
	Macrogastra plicatula (Draparnaud 1801)	Wittnau, Switzerland	47° 25' 54.21" N, 8° 01' 06.15" E	801	06.08.2012	1	1x22
	Macrogastra ventricosa (Draparnaud 1801)	Rottenburg am Neckar, Germany	48° 27' 55.81" N, 8° 55' 12.57" E	383	04.09.2011	1	1x25
	Cochlodina laminata (Montagu 1803)	Rottenburg am Neckar, Germany	48° 27' 55.81" N, 8° 55' 12.57" E	383	04.09.2011	3	3x25
	Cochlodina fimbriata (Rossmässler 1835)	Benkerjoch/Wittnau, Switzerland<br>Wittnau, Switzerland	47° 26' 20.78" N, 7° 59' 02.34" E<br>47° 25' 54.21" N, 8° 01' 06.15" E	868<br>801	17.09.2011<br>06.08.2012	3	2x25<br>1x25
	Balea perversa (Linnaeus 1758)	Frösslunda, Öland, Sweden	56° 33' 21.30" N, 16° 34' 23.76" E	10	08.10.2011	3	3x25
	Balea biplicata (Montagu 1803)	Rottenburg am Neckar, Germany	48° 27' 55.81" N, 8° 55' 12.57" E	383	04.09.2011	3	3x25
Bothriembryontidae	*Discoleus aguirrei* (Doering 1884)	Bahía Blanca, Argentina	38° 35' 08.93" S, 62° 04' 36.13" W	106	12.08.2011	3	3x25
	Discoleus ameghinoi (von Ihering 1908)	Sierra Grande, Argentina	41° 38' 22.00" S, 65° 01' 29.00" W	9	28.11.2011	3	3x25
Odontostomidae	*Plagiodontes patagonicus* (d'Orbigny 1835)	Bahía Blanca, Argentina	38° 40' 40.96" S, 62° 16' 30.06" W	44	05.08.2011	3	3x25
	Cyclodontina (Ventania) avellanedae (Doering 1881)	Sierra de la Ventana, Argentina	38° 04' 09.00" S, 62° 01' 37.00" W	463	15.10.2011	3	3x25
Strophocheilidae	*Austroborus lutescens dorbignyi* (Doering 1876)	Sierra de la Ventana, Argentina	38° 04' 09.00" S, 62° 01' 37.00" W	463	15.10.2011	3	3x25
Discidae	*Discus rotundatus* (Müller 1774)	Benkerjoch/Wittnau, Switzerland<br>Chienberg bei Grindel, Switzerland	47° 25' 54.21" N, 8° 01' 06.15" E<br>47° 23' 07.99" N, 7° 31' 26.39" E	801<br>715	17.09.2011<br>11.09.2011	3	1x25, 1x24<br>1x17
Oxychilidae	*Oxychilus navarricus helveticus* (Blum 1881)	Benkerjoch/Wittnau, Switzerland	47° 25' 48.47" N, 8° 00' 18.98" E<br>47° 25' 54.21" N, 8° 01' 06.15" E	809<br>801	17.09.2011<br>17.09.2011	2	1x25<br>1x25
	Oxychilus draparnaudi (Beck 1837)	Sartrouville, France	48° 55' 52.24" N, 2° 08' 54.80" E	31	17.08.2012	3	3x25
	Aegopinella nitens (Michaud 1831)	Benkerjoch/Wittnau, Switzerland<br>Wittnau, Switzerland	47° 26' 09.02" N, 8° 01' 24.86" E<br>47° 25' 54.21" N, 8° 01' 06.15" E	764<br>801	17.09.2011<br>06.08.2012	3	1x25<br>2x25
Zonitidae	*Zonitoides nitidus* (Müller 1774)	Istein, Germany	47° 39' 45.55" N, 7° 31' 27.09" E	234	30.04.2012	3	3x25
Limacidae	*Limax maximus* Linnaeus 1758	Lörrach, Germany	47° 36' 36.16" N, 7° 38' 52.06" E<br>47° 36' 38.70" N, 7° 38' 55.73" E<br>47° 36' 38.70" N, 7° 38' 55.73" E	355<br>340<br>340	17.10.2011<br>18.05.2012<br>08.08.2012	3	1x25<br>1x25<br>1x25
	Limax tenellus Müller 1774	Benkerjoch/Wittnau, Switzerland	47° 25' 54.21" N, 8° 01' 06.15" E<br>47° 26' 09.02" N, 8° 01' 24.86" E	801<br>764	17.09.2011<br>17.09.2011	3	1x25<br>1x25

			47° 25' 48.47" N, 8° 00' 18.98" E	809	17.09.2011		1x25
	Limax cinereoniger Wolf 1803	Maulburg, Germany	47° 39' 24.02" N, 7° 47' 15.83" E	526	19.05.2012	2	2x25
Agriolimacidae	*Deroceras reticulatum* (Müller 1774)	Torslunda, Öland, Sweden	56° 36' 57.31" N, 16° 29' 54.72" E	50	07.10.2011	3	3x25
Vitrinidae	*Vitrina pellucida* (Müller 1774)	Torslunda, Öland, Sweden	56° 36' 57.31" N, 16° 29' 54.72" E	50	07.10.2011	1	1x25
	Vitrinobrachium breve (Férussac 1821)	Susch, Switzerland	46° 44' 54.12" N, 10° 05' 11.99" E	1477	27.08.2012	1	1x25
Arionidae	*Arion (ater) rufus* (Linnaeus 1758)	Rottenburg am Neckar, Germany	48° 25' 25.93" N, 8° 57' 10.79" E	524	21.07.2012	3	3x25
	Arion vulgaris (Moquin-Tandon 1855)	Reinach, Switzerland	47° 30' 35.62" N, 7° 35' 25.46" E	357	01.07.2012	1	1x25
	Arion distinctus (Mabille 1868)	Benkerjoch/Wittnau, Switzerland	47° 25' 54.21" N, 8° 01' 06.15" E	801	17.09.2011	1	1x25
Helicidae	*Helix pomatia* Linnaeus 1758	Lörrach, Germany	47° 36' 41.70" N, 7° 38' 19.33" E	459	12.06.2012	3	3x25
	Cepaea nemoralis (Linnaeus 1758)	Lörrach, Germany	47° 36' 37.86" N, 7° 38' 52.94" E	350	17.06.2012	3	3x25
	Cepaea hortensis (Müller 1774)	Viersen, Germany	51° 16' 16.66" N, 6° 22' 10.05" E	62	15.07.2012	3	3x25
	Cepaea vindobonensis (Férrusac 1821)	Esztergom, Hungary	47° 46' 43.33" N, 18° 43' 15.95" E	105	04.08.2011	3	3x25
	Cornu aspersum (Müller 1774)	Siteia, region of Lasithi, Crete, Greece	35° 12' 31.24" N, 26° 06' 18.91" E	20	02.10.2012	3	3x25
	Eobania vermiculata (Müller 1774)	Vinisce, Croatia	43° 29' 02.00" N, 16° 06' 45.00" E	2	01.08.2012	3	3x25
	Theba pisana (Müller 1774)	Saint-Clément-des-Baleines, France	46° 13' 31.10" N, 1° 32' 38.08" W	2	13.07.2012	3	3x25
	Arianta arbustorum (Linnaeus 1758)	Gantrisch, Switzerland	46° 42' 18.70" N, 7° 26' 42.10" E	1800	22.05.2011	3	3x25
	Helicigona lapicida (Linnaeus 1758)	Rottenburg am Neckar, Germany	48° 27' 55.81" N, 8° 55' 12.57" E	383	04.09.2011	3	3x25
	Isognomostoma isognomostomos (Schröter 1784)	Wittnau, Switzerland	47° 25' 54.21" N, 8° 01' 06.15" E	801	06.08.2012	3	3x25
Bradybaenidae	*Fruticicola fruticum* (Müller 1774)	Kleinkems, Germany	47° 40' 37.05" N, 7° 31' 14.24" E	230	20.06.2011	3	3x25
Cochlicellidae	*Cochlicella acuta* (Müller 1774)	Honfleur, France	49° 24' 38.64" N, 0° 09' 55.33" E	1	18.08.2012	3	3x25
Helicodontidae	*Helicodonta obvoluta* (Müller 1774)	Arlesheim, Switzerland	47° 29' 17.23" N, 7° 37' 55.94" E	480	04.07.2011	3	3x25
Hygromiidae	*Helicella itala* (Linnaeus 1758)	Aesch, Switzerland	47° 29' 04.83" N, 7° 36' 23.72" E	290	02.08.2011	3	3x25
	Candidula intersecta (Poiret 1801)	Texel, the Netherlands	53° 04' 01.32" N, 4° 43' 34.60" E	5	01.07.2011	1	1x25
	Xerolenta obvia (Menke 1828)	Susch, Switzerland	46° 44' 52.83" N, 10° 04' 51.76" E	1440	27.08.2012	3	3x25
	Monachoides incarnatus (Müller 1774)	Chienberg bei Grindel, Switzerland	47° 23' 07.99" N, 7° 31' 26.39" E	715	11.09.2011	1	1x25
	Trochulus villosus (Studer 1789)	Gurnigel, Switzerland	46° 45' 08.06" N, 7° 27' 09.63" E	1330	25.04.2011	3	3x25
	Trochulus sericeus (Draparnaud 1801)	Wittnau, Switzerland	47° 25' 54.21" N, 8° 01' 06.15" E	801	06.08.2012	2	2x25
	Monacha cartusiana (Müller 1774)	Aesch, Switzerland	47° 28' 08.10" N, 7° 36' 18.60" E	300	15.07.2012	3	3x25

[1] Sample size: number of gastropods and number of sperm used in the data analyses.
