# Supplementary material for: Breeding system, shell size and age at sexual maturity affect sperm length in stylommatophoran gastropods
Source: BMC Evol Biol. 2016 Apr 29;16:89. doi: 10.1186/s12862-016-0661-9 (PMC4850656; doi:10.1186/s12862-016-0661-9)
Supplement: Additional file 4: — GeneBank accession numbers for the new nucleotide sequences. (PDF 9 kb) [file 12862_2016_661_MOESM4_ESM.pdf]

Additional file 4: GenBank accession numbers for the new nucleotide sequences.

28S_RNA_new.sqn	*Aegopinella nitens*	KT371378
28S_RNA_new.sqn	*Arion vulgaris*	KT371379
28S_RNA_new.sqn	*Austroborus dorbigny*	KT371380
28S_RNA_new.sqn	*Candidula intersecta*	KT371381
28S_RNA_new.sqn	*Cepaea vindobonensis*	KT371382
28S_RNA_new.sqn	*Chondrina clienta*	KT371383
28S_RNA_new.sqn	*Clausilia rugosa*	KT371384
28S_RNA_new.sqn	*Cochlodina fimbriata*	KT371385
28S_RNA_new.sqn	*Columella columella*	KT371386
28S_RNA_new.sqn	*Cyclodontina avellanedae*	KT371387
28S_RNA_new.sqn	*Discoleus aguirrei*	KT371388
28S_RNA_new.sqn	*Discoleus ameghinoi*	KT371389
28S_RNA_new.sqn	*Limax cinereoniger*	KT371390
28S_RNA_new.sqn	*Limax maximus*	KT371391
28S_RNA_new.sqn	*Limax tenellus*	KT371392
28S_RNA_new.sqn	*Macrogastra ventricosa*	KT371393
28S_RNA_new.sqn	*Monachoides incarnatus*	KT371394
28S_RNA_new.sqn	*Orcula dolium*	KT371395
28S_RNA_new.sqn	*Oxychilus draparnaudi*	KT371396
28S_RNA_new.sqn	*Oxychilus navarricus*	KT371397
28S_RNA_new.sqn	*Plagiodontes patagonicus*	KT371398
28S_RNA_new.sqn	*Pyramidula pusilla*	KT371399
28S_RNA_new.sqn	*Trochulus sericeus*	KT371400
28S_RNA_new.sqn	*Trochulus villosus*	KT371401
28S_RNA_new.sqn	*Vertigo pygmaea*	KT371402
28S_RNA_new.sqn	*Vitrinobrachium breve*	KT371403
28S_RNA_new.sqn	*Xerolenta obvia*	KT371404
COI_new.sqn	*Arion vulgaris*	KT371405
COI_new.sqn	*Austroborus dorbigny*	KT371406
COI_new.sqn	*Balea biplicata*	KT371407
COI_new.sqn	*Balea perversa*	KT371408
COI_new.sqn	*Cepaea vindobonensis*	KT371409
COI_new.sqn	*Clausilia rugosa*	KT371410
COI_new.sqn	*Cochlodina fimbriata*	KT371411
COI_new.sqn	*Cochlodina laminata*	KT371412
COI_new.sqn	*Cyclodontina avellanedae*	KT371413
COI_new.sqn	*Discoleus aguirrei*	KT371414
COI_new.sqn	*Discoleus ameghinoi*	KT371415
COI_new.sqn	*Isognomostoma isognomostomos*	KT371416
COI_new.sqn	*Macrogastra plicatula*	KT371417
COI_new.sqn	*Macrogastra ventricosa*	KT371418
COI_new.sqn	*Oxychilus draparnaudi*	KT371419
COI_new.sqn	*Oxychilus navarricus*	KT371420
COI_new.sqn	*Plagiodontes patagonicus*	KT371421
COI_new.sqn	*Pyramidula pusilla*	KT371422
COI_new.sqn	*Vertigo pygmaea*	KT371423
COI_new.sqn	*Vitrinobrachium breve*	KT371424
COI_new.sqn	*Xerolenta obvia*	KT371425
